# Geographical accessibility and spatial coverage modelling of public health care network in rural and remote India

**DOI:** 10.1371/journal.pone.0239326

**Published:** 2020-10-21

**Authors:** Veenapani Rajeev Verma, Umakant Dash

**Affiliations:** Department of Humanities and Social Sciences, Indian Institute of Technology Madras (IIT M), Tamil Nadu, Chennai, India; Institute of Economic Growth, INDIA

## Abstract

**Background:**

Long distances to facilities, topographical constraints, inadequate service capacity of institutions and insufficient/ rudimentary road & transportation network culminate into unprecedented barriers to access. These barriers gets exacerbated in presence of external factors like conflict and political disruptions. Thus, this study was conducted in rural, remote and fragile region in India measuring geographical accessibility and modelling spatial coverage of public healthcare network.

**Methods:**

Vector and raster based approaches were used to discern accessibility for various packages of service delivery. Alternative scenarios derived from local experiences were modelled using health facility, population and ancillary data. Based on that, a raster surface of travel time between facilities and population was developed by incorporating terrain, physical barriers, topography and travelling modes and speeds through various land-cover classes. Concomitantly, spatial coverage was modelled to delineate catchment areas. Further, underserved population and zonal statistics were assessed in an interactive modelling approach to ascertain spatial relationship between population, travel time and zonal boundaries. Finally, raster surface of travel time was re-modelled for the conflict situation in villages vulnerable to obstruction of access due to disturbed security scenario.

**Results:**

Euclidean buffers revealed 11% villages without ambulatory & immunization care within 2 km radius. Similarly, for 5 km radius, 11% and 12% villages were bereft of delivery and inpatient care. Travel time accessibility analysis divulged walking scenario exhibiting lowest level of accessibility. Enabling motorized travel improved accessibility measures, with highest degree of accessibility for privately owned vehicle (motorcycle and cars). Differential results were found between packages of services where ambulatory & immunization care was relatively accessible by walking; whereas, delivery and inpatient care had a staggering average of three hours walking time. Even with best scenario, around 2/3rd population remained unserved for all package of services. Moreover, 90% villages in conflict zone grapples with inaccessibility when the scenario of heightened border tensions was considered.

**Conclusions:**

Our study demonstrated the application of GIS technique to facilitate evidence backed planning at granular level. Regardless of the scenario, the analysis divulged inaccessibility to delivery and inpatient care to be most pronounced and majority of population to be unserved. It was suggested to have concerted efforts to bolster already existing facilities and adapt systems approach to exploit synergies of inter-sectoral development.

## Introduction

Universal Health Coverage(UHC) has become the focal point of health policy discourse as the world has made transition from Millennium Development Goals(MDG s) to Sustainable Development Goals (*Goal 3*.*8*: *“Achieve UHC*, *including financial risk protection*, *access to quality essential healthcare services and access to safe*, *effective*, *quality and affordable essential medicines and vaccines for all*”). Access to healthcare which is central to UHC is intractable concept and has multiple definitions with respect to ability to get care, act of seeking care, actual delivery of care, indicators thereof and is context specific. The framework of *effective coverage* propounded by Tanahashi encompasses the domain of accessibility (spatial and non-spatial) as a component in ascertaining barriers to Universal Health Coverage [1]. Accessibility is also one of the themes pertaining to access and utilization embedded in Penchansky’s conceptual framework of availability, accessibility, affordability, acceptability and accommodation [2] where spatial accessibility is captured in first two components. Accessibility can be defined as the factors intervening between the perception of need and realization of utility [3]. Effective accessibility to medical services reflects an individual/ family’s ability, mobility and time to reach a service once need has been established by a potential health service user [4], which can be distinguished from *potential accessibility* which simply implies the existence of a service, regardless of whether it is *effectively accessible*. Spatial accessibility measuring travel impedance (distance or time) between patient location and service points impacts the progression from potential to realized access.

Geographic Information Systems are one of the suite of information and communication technology enabled solution recommended by World Health Organization (WHO) and Asian Development Bank (ADB) to address system resiliency and universal health coverage inefficiencies [5]. Geospatial data is directly relevant to all three main functions of country’s public health system: monitoring community health & identification of health problems & priorities; ensuring universal access to appropriate and cost-effective care; and policy making to solve local and national health problems [6]. Most published measures of spatial accessibility to healthcare can be classified more simply into four categories: provider-population ratios, distance to nearest provider, average distance to set of providers, and gravitational models of provider influence [7]. However, there is a major lacunae with respect to studies exploring the dimension of geographic accessibility and even more scarce literature delving into spatial modelling using appropriate techniques to the setting. In Indian context specifically, the issue of distance- time as barriers to healthcare services hasn’t been analyzed systematically and the digital cartography has been used only for visualization of health indicators rather than informed decision making.

Taxonomy for healthcare studies categorizes the study of spatial accessibility as the study of spatial potential which is studied by both distance and time approach. Straight line distance however, are not symptomatic to true provider accessibility due to varied road distance, quality, terrain and seasonal variation and overestimate the population that is within an hour of health facility [8]. Thus, using transport network elevation and other natural barriers can provide more accurate estimates. Literature however, indicates relative efficiency of raster based techniques vis. a vis. network analysis specifically in areas with rudimentary/dilapidated infrastructure and road network. The study by Delamater [9]; revealed raster based method identifying more total area, zip codes and population as being underserved than network method as raster method produced fewer unique contiguous area than network method. Raster based analysis by incorporating both network and off-network modes of travel and allowing for complex array of barriers redresses the limitation of straight- line distance model and network analysis, and is thus, preferred method of analysis in remote, rural and topographically challenging settings. Therefore, our study has strived to adopt a comprehensive and holistic approach to model spatial accessibility and population coverage in the area setting. Current study has attempted to provide a succinct analysis via multimodal modelling to gauge proximity and coverage using both Euclidean distance and Raster based approach. The geographic access and spatial coverage surfaces produced in our analysis provide visually powerful tools that can be used to support health research and decision making for planning and resource allocation at district level, thereby aiding in solving location-allocation conundrum. The rationale of conducting this study is to demonstrate significant spatial variations in geographical accessibility and spatial coverage of health system across different travel scenarios. The quintessential feature of this approach is to incorporate information on demand and supply of care in order to support health planners in identifying potential locations for new health facilities where maximum increase in accessibility can be achieved. To our knowledge, this is the first study from India exploring different packages of healthcare services as a geographical measure of Indicator 3.8.1(Coverage of Essential Health Services) of Goal 3, Sustainable Development Goals. Existing studies only delves into single health service or are based on assumption that any service in the health facility network can be chosen if it is reachable within specified travel time which is an unrealistic assumption as service provisioning and readiness amongst facilities is very heterogeneous in Indian setting. Hence, our study is an improvisation over previous literature as separate analysis has been conducted by subdividing health facilities and population grid in tandem with the type of service being investigated. We conducted a detailed service availability mapping of entire public health facility network in our study area enabling the identification of facilities providing specific services. Another novelty of our study arises from holistic methodological approach of incorporating household survey database allowing for participatory, dynamic and interactive approach of parametrization of travel scenarios, mode of transportation and travel speeds using actual utilization data and individual experiences by users, whereas, current literature is constrained by availability of accurate input data and limitations are inherent in the assumptions and parameterizations. Improving the accuracy and relevance in this context requires greater accessibility to, and flexibility in, travel time modelling tools to facilitate the incorporation of local knowledge and rapid exploration of multiple travel scenarios [10]. Incorporating such local knowledge refines the estimates as the travelling time and extent of catchment areas are sensitive to the mode of transportations and travelling speeds. Additionally, we have also collated and synthesized the administrative data in our framework aiding estimation of the population coverage. Finally, geographical accessibility in difficult settings like rural, remote and conflict zones poses formidable challenges to the users. Specifically, population residing in the contested borderlands have to reckon with peculiar security threats as episodes of firing and shelling obstructs the physical access further. Yet, there is an absence of representation of difficult settings in the literature and spatial accessibility to reach target population in fragile zones is not explored, which we have attempted in present study.

## 2. Methods

### 2.1 Study area

**Fig 1:**
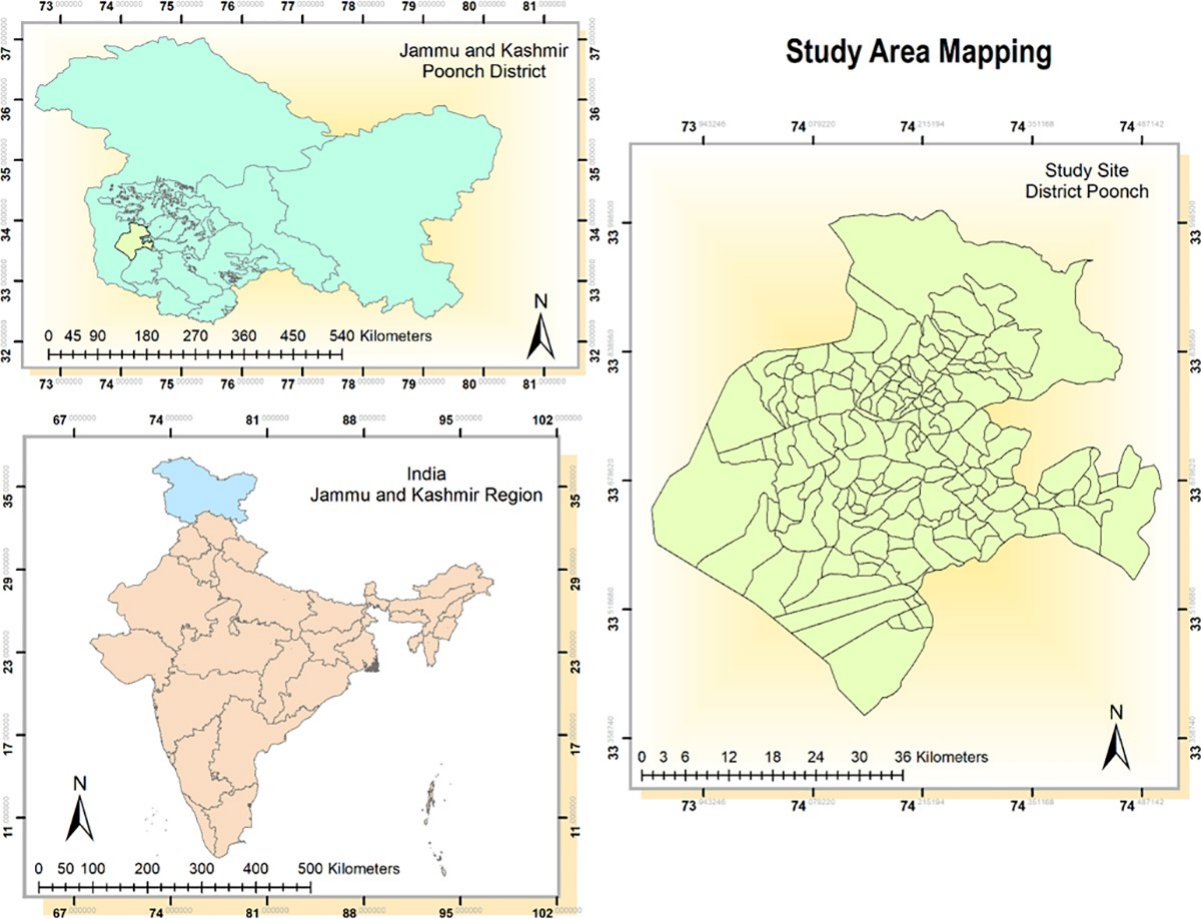
Map of the study site.

The study was conducted in Poonch district which is the remotest district of erstwhile state of Jammu and Kashmir (now a Union Territory) in India. The topography of district is hilly and mountainous barring few low lying valleys with Pir Panjal range separating it from Kashmir region. It is nestled between 33°25' to 34°01' north latitude and 73°58' to 74°35' east longitude and is surrounded by Kashmir valley (Baramulla, Budgam, Shopian and Kulgam districts) in the north-east, district Rajouri in the south and Pakistan administered Kashmir in the west (Fig 1). The altitude of region varies from 1007 m to 4700 m above the sea level and is susceptible to inclement weather conditions with snow-cover during winters. More than half (56.81%) of total area in the district is covered by forests and the wetlands in the area predominantly comprising of Poonch river and its tributaries. According to 2011 census, district has population of 476,820 and an area spanning 1674 square kilometer with population density of 285 per square kilometer. The region has literacy rate of 68.60% and has more than one third of tribal population. Also, the district is relatively impoverished with one third population sustaining below poverty line.

The area is encumbered with heavy military deployment as it is bounded by Line of Control (porous boundary between Indian and Pakistani administered Kashmir and is one of the most complex frontier systems in the world) bearing the brunt of cease fire violations and conflict. The district is also low performing in terms of health indicators and has been categorized as *high priority district* by Government of India based on poor performance in health composite index [11]. The district is divided into three medical blocks viz. Mandi, Surankote and Mendhar. There are insurmountable healthcare access barriers in region which is reflective in suboptimal health facility coverage. The border is fenced as a fallout of armed militancy and infiltration of militants via porous borders, and at numerous places fencing is done much inside zero line enclosing vast swathes of population and these lands are fenced and gated ensuing obstructions to access. Some of the health facilities and catchment population are inside this zero line which has been included in the study.

### 2.2 Study design

**Fig 2:**
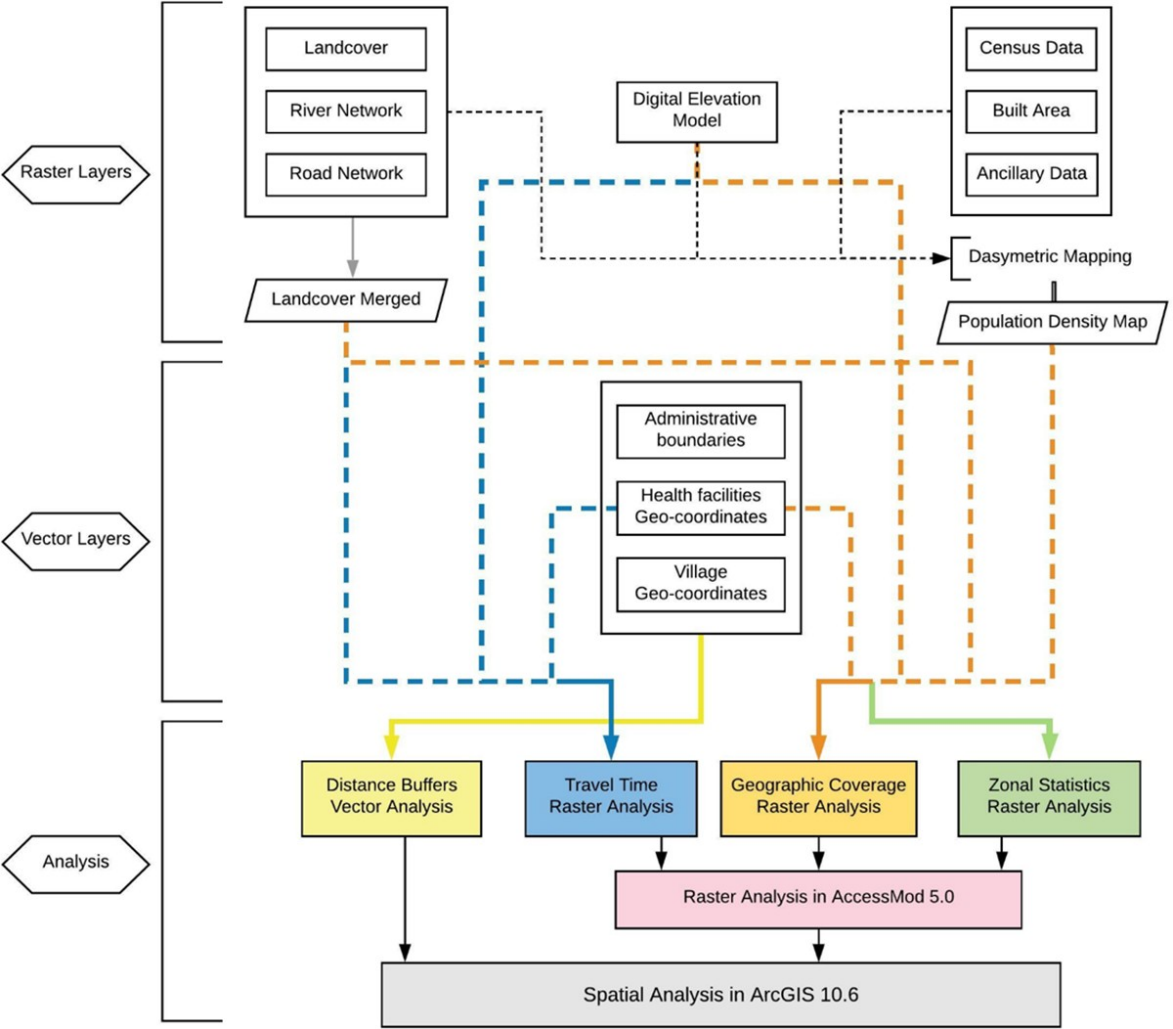
Flow chart of methodology.

The study employed amalgamation of vector and raster approaches to discern geographical accessibility and spatial coverage in the study area (see Fig 2). The analysis was conducted using myriad of alternative scenarios incorporating various package of healthcare delivery i.e. i) ambulatory care ii) inpatient care iii) delivery care and iv) childhood immunization. Spatial data of public health facilities was obtained by conducting facility survey collecting geo-coordinates of facilities in the study site. In conjunction with in-situ data collection, secondary data for geospatial modelling was collated from multitude of sources ranging from administrative data to satellite based remote sensing imageries. Geoprocessing models for this analysis included i) Creation of Euclidean distance buffer zones (generating catchments at physical distances away from village centroid) in two dimension Cartesian plane using vector spatial analysis. ii) Performing Spatial overlay analysis allowing the identification of relationship between the location of health facilities and village centroids to ascertain availability and adequacy of health facilities within the zones. iii) Developing Raster based travel time cost surface model to examine the spatial distribution of travel time towards nearest health facility (ies). Modelling of various alternative travel scenarios simulating disparate primary transportation modes was exercised to capture accessibility to these facilities. iv)Conducting geographic coverage analysis allowing for integration of spatial distribution of health services and population to gauge the convergence of supply and demand and assessing whether physically accessible facilities have adequate capacity to cover such demand. v) Computing Zonal statistics to unravel population accessibility coverage at the levels of zones (medical blocks) via interactive modelling of travel time distribution, spatial distribution of target population and boundaries of zones. Legion of parameters such as land use/ land cover, gradient in elevation, road network, physical barriers, spatial distribution of village settlements, location of existing health facilities were modeled in GIS domain using AccessMod 5.0 software package developed by World Health Organization **[12].** Further, Spatial analysis of raster output was done in ArcMap 10.6 divulging the spatial relationship between the population, travel time and zonal boundaries.

### 2.3 Datasets

This section summarizes the data source, spatial resolution and other specification details of input geospatial data used for modelling travel time and geographic coverage in the region. All input datasets were clipped to the administrative boundaries of the district, as derived from NASA’s Socioeconomic Data and Applications Center. Data layers were projected consonantly into the spatial reference frame, WGS84/UTM Zone 44 N and all rasters used in travel time analysis were attuned specifically at 30-mtrs resolution.

#### 2.3.1 Healthcare facilities

Health facilities represented in study site are stratified as: i) Second tier District hospital and Community health centers(DH and CHCs) providing secondary healthcare ii) First tier Primary health centers(PHCs) serving as first point of contact between population and qualified doctors and iii) Sub-centers operating as peripheral health institution available to rural population. The health system of entire district comprises of 137 subcenters, 44 primary health centers (including upgraded), 3 community health centers and 1 district hospital. All of these facilities provide ambulatory and immunization care, whereas, only 34 and 28 facilities are equipped for service provisioning of delivery and inpatient related services. Geographic coordinates of all the facilities (accuracy of ± 10 meters) were collected by conducting field visit to these facilities. A vector layer of spatial location of facilities was created which was superimposed into final land-cover grid. One facility was located on the cell considered to be waterbody, which was then, manually moved to nearest cell.

#### 2.3.2 Merged land cover distribution grid

Land use and land cover mapping for 2017–18 on 1:250K scale using multi-temporal Resourcesat- 2 terrain corrected Linear Imaging Self Scanning Sensor (LISS-III) satellite data was obtained from National Remote Sensing Center, Indian Space Research Organization upon request. This raster dataset is constructed by conflating 3 seasons–Monsoon–Kharif: August-October, Post Monsoon–Rabi: December–March and Pre Monsoon–Zaid: April–May. The land-cover data comprised 18 land cover classes which was reclassified into 7 major generic classes—i) Forest land ii) Grassland iii) Cropland iv) Settlement/Built area v) Wetlands vi) Waste-land/Fallow/Other land-cover and vii) Snowcapped land. *Road network dataset*, obtained from Open Street Map and further digitized using Google Earth was reclassified into road classes encompassing i) National Road ii) Secondary Roads and iii) Local tertiary Roads. Tertiary roads were further prorated into i) Four wheeler passable roads ii) Two wheeler passable roads and iii) Walking only roads. The data was rectified to connect segments of roads omitted through digitization and deleting those extending into waterbodies. *Rivers and waterbodies* mask embodying physical barriers was extracted from Land use and land cover map. Both road network and river dataset were rasterized to 30 meters gridded cells and were then superimposed on land-cover raster dataset to create merged land-cover dataset with 12 unique land feature classes.

#### 2.3.3 Digital elevation model

It is pertinent to consider DEM for analyzing movement of patients across varying topography. The DEM is used as the reference grid for each individual analysis and the extent, projection and resolution of all other layers used in the project hinges upon DEM. Raster format layer of Digital elevation model (DEM) extracted from CartoSAT-1, a high resolution satellite data was retrieved from Bhuvan website [13] for 2.5 m spatial resolution in track stereo. The resolution of raster layer was then changed to 30m using resampling technique of Cubic convolution interpolation.

#### 2.3.4 Population distribution grid

The gridded population distribution of 1 hectare estimated in continuous raster surface was created by dasymetric modelling approach. The design conflated detailed census estimates for 2015 (Projected from Indian census, 2011) and widely available, remotely sensed and geospatial ancillary data in a flexible Random Forest estimation technique (model version 2c) following methodology described in **[14]** and [15]. A Random Forest algorithm was used to generate gridded population density estimates that were subsequently used to dasymetrically disaggregate population counts from administrative units into grid cells of 1 hectare spatial resolution. Population density response variable and legion of covariates were computed at administrative level which was then used to fit Random Forest model for predicting population density at grid cell level (generating dasymetric weighting layer in conjunction with covariates coalescing from following ancillary data–i) LULC raster layer at 30 m spatial resolution ii) Lights at night raster data at 15 arc- second derived from imagery collected by Suomi National Polar-orbiting Partnership (NPP) Visible Infrared Imaging Radiometer Suite (VIIRS) sensor iii) Mean annual temperature and mean annual precipitation raster data from WorldClim/BioClim 1950–2000 at 30 arc-second, mosaicked and subset to match extent of land-cover data of the region iv) Buildings, residential and infrastructure vector layer from Open Street Map v) Protected areas vector layer from World Database on Protected Areas vi)River and waterbodies network vector layer vii) Road network vector layer viii) Built-area raster layer ix) Elevation and derived slope raster layer. These collated set of covariates were used for model fitting and prediction of the final layer.

### 2.4 Parameters

#### 2.4.1 Travelling scenario

For estimating the physical accessibility to healthcare network, it is imperative to incorporate the mode of transportation and speed of travel by patient as they navigate through varying topographical variations and landscape features. Limited road networks, poor road quality and ardous terrain induces population to use diverse form of transportation to reach health facilites. Simulating use of primary transportation modes in real life, four different travel scenarios were specified(walking, motorcycle, public transport and car). These modes were ascertained from household survey which was administered by authors around the same time of facility survey(Additional dataset 2, Table 2). The rationale of household survey was to collect information pertaining to household’s socio-economic status, and individual’s morbidity status, access/utilization of healthcare services and healthcare expenditure on ambulatory, immunization, inpatient and delivery care. The questionnaire encompassed questions such as: ‘How much distance did you travel to reach the point of care?’ ‘Which transportation mode did you use to reach the point of care?’ ‘How much time did it take to reach the point of care?’. These questions were used to capture information about patient’s point of origin, destination, transportation media and time taken to reach destination. On the basis of which, following scenarios were considered: a) ***Scenario 1***: Walking only b) ***Scenario 2***: Walking & Motorcycle c) ***Scenario 3*:** Walking and Public Transport and d) ***Scenario 4*:** Walking and Privately owned 4 wheeler. While travel speed for walking was adapted from Global Accessibility Map, 2015 [16], a more evidence based and participatory data from household survey was used to gauge speed in motorized scenarios allowing for knowledge on local travel habits derived through lived experience. Such analysis effectively capture useful range of travel time scenarios allowing greater transparency around model assumptions. The details of scenarios and corresponding speeds are illustrated in Table 1. For analytical purposes, rivers and wetlands were conceded to be impassable to any form of transportation. Further, Anisotropic i.e. slope dependent approach was used in analysis for walking corrections. AccessMod uses Digital Elevation Model to compute slopes that are in turn used to modify speed of travel indicated in travel scenarios. Correction is made using Tobler’s formula which increases or decreases effective walking speed analogous to steepness of slope through following formula:

$$V=V_{F}\;*\;e^{‐3.5*abs\left(S+0.05\right)}$$

Where V is corrected walking speed in kms/hr, V_F_ is walking speed on a flat surface and S is slope in hundredth of percent.

**Table 1:** Travel scenario representing travel speeds, per land-cover type.

Travel Speeds(km/hr)	Scenario 1	Scenario 2		Scenario 3		Scenario 4	
Land-cover Type	Walking	Walking	Motorcycle	Walking	Public Transport	Walking	Private vehicle
Forestland	1.67	1.67	--	1.67	--	1.67	--
Grassland/Plantation	1.67	1.67	--	1.67	--	1.67	--
Cropland	1.67	1.67	--	1.67	--	1.67	--
Settlement	2.5	2.5	--	2.5	--	2.5	--
Wasteland/Fallow/Other land-cover	2.5	2.5	--	2.5	--	2.5	--
Waterbodies	NA	NA	--	NA	--	NA	--
Snowcapped land	1.25	1.25	--	1.25	--	1.25	--
Major roads	2.5	--	40	--	1.25	--	40
Secondary roads	2.5	--	25	--	40	--	35
Tertiary roads: passable to all transportation	2.5	--	15	--	20	--	15
Tertiary roads: passable to motorcycle/bicycle	2.5	--	15	2.5	--	2.5	--
Tertiary roads: walking only	2.5	2.5	--	2.5	--	2.5	--

#### 2.4.2 Population coverage capacity

Population coverage capacity of each facility tantamount to maximum number of people the service can cover over a particular period of time based on its patient’s capacity. It corresponds to extent to which health facilities are utilized by patients compared to total number of patients health facility can potentially serve. Population coverage capacity of health institutions should be linked with maximum time taken for patient to access these facilities for delineation of catchment areas. Data from multitude of sources such as administrative records, hospital records health management information systems and facility survey was extracted to determine capacity and has been provided in Additional dataset 1.

Total population served by Subcenters and Primary health centers was ciphered by following formula as indicated in AccessMod 5.0 manual:

Totalpopulationserved=(No.ofhealthworkers*No.ofpatientsseeninoneaverageday*No.ofdaysworkedperyear)/(Averageno.ofoutpatientvisitspercapitaperyear)

Further, for community health centers and district hospital, parameter was computed as:

Totalpopulationserved=(No.ofbeds*Occupancyrate*No.ofworkingdaysinayear)/(Averageno.ofinpatientadmittancespercapitaperyear*Averagelengthofstay)

### 2.5 Spatial modelling

*Feature-based Proximity analysis* measuring physical accessibility was done by creating two-ring buffer around village location in ArcGIS 10.6. This was done by employing feature based proximity tool to create vector polygons at two varied specified buffer distance enclosing spatial location of village centroids. Euclidean buffers were chosen over Geodesic buffers as features were concentrated in a relatively small area in projected coordinate system. The buffers circumscribed 2 km and 5 km distance for ambulatory and immunization care and encompassed a distance of 5 km and 10 km for delivery points and inpatient care. Point layer of facilities was overlaid on village boundaries shapefile and spatial join was performed to discern availability of facilities delivering services within the zone.

*Accessibility analysis* estimating minimum amount of time required for population to travel to nearest health facility was conducted in AccessMod 5.0. This modelling program uses a least cost path(friction surface) approach to produce raster layer across target area where each gridded cell represent minimum travel time from cell’s location to target destination. The analysis accommodates landscape constraints and travelling scenario table defining travel speeds for each merged landcover class. For this accessibility analysis, maximum travel time was set to zero in order to compute travel time for full extent of study area and each scenario-specific travel time raster layer was further reclassified and converted to four incremental travel time zones (within an hour to more than 3 hours) as polygon vectors. Time taken for patient to reach a health facility from his/her household isn’t necessarily equal to time taken for that same person to perform return journey. The results are sensitive to the choice of movement as topography is pronounced in our setting, hence, anisotropic analysis was done to estimate the travel *towards* facilities.

*Geographic coverage analysis* defining catchment area associated with each facility within travel-time zone and assessing proportion of unserved population was undertaken. This analysis takes into account availability of services (i.e. capacity of health facilities to attend to patients) subjected to physical accessibility constraints. Input data subsumed health facilities vector layer, travel time distribution grid, population distribution grid, land-cover grid, travelling scenario table, digital elevation model layer and health facilities coverage capacity table enabling the modelling exercise. 1.2% population was found to be distributed on barriers, hence, population correction tool embedded in AccessMod 5.0 was employed to adjust population distribution raster so that population found in barrier pixels is redistributed outside of these barriers. Maximum travel time for patient needing to access health facility typically depend on type of care and severity of condition. A travel time of 1 hour for ambulatory and immunization services and 2 hours for delivery and inpatient care was specified in order to delineate the catchment areas.

#### Ethics approval and consent to participate

The protocol for this study has been approved by ethical committee of Indian Institute of Technology Madras. Official permissions were obtained from Directorate of Health and Family Welfare, Jammu and Kashmir and district health authorities to conduct facility survey.

## 3. Results

### 3.1 Proximity analysis

**Fig 3:**
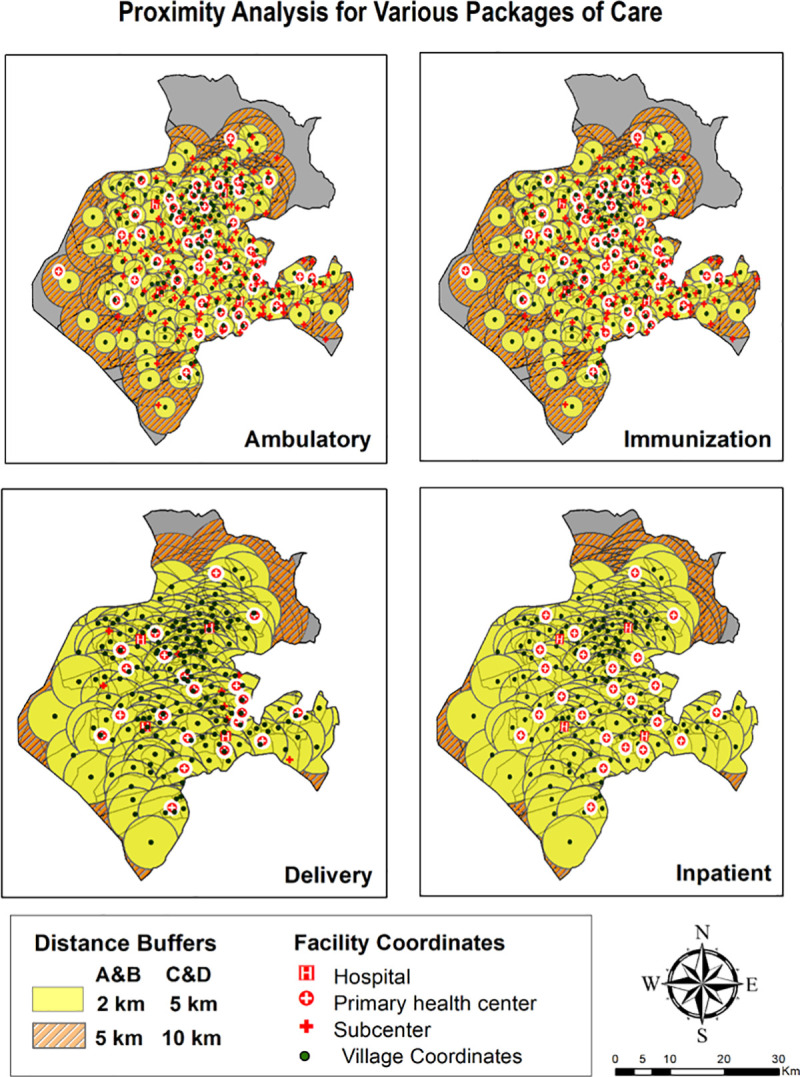
Feature based proximity analysis.

Euclidean buffers encapsulating the simplest method of proximity analysis by generating catchment at physical distances from village centroids are illustrated in Fig 3. Overlay of facility layer highlighted the presence and count of facilities within buffer region. Catchment areas indicated by circles of two radii defining service areas for ambulatory and immunization care suggested adequate services. The analysis can be corroborated with findings surmised in Table 2 revealing that only 11.11% villages didn’t have ambulatory care provisioning within 2 km radius. However, extending the radius to 5 km, reduces the proportion of villages with no coverage to 0.5%. To explicate the proximity to delivery points and inpatient care, a distinct buffer distance of 5 km and 10 km revealed the percentage of villages with no coverage as analogous to ambulatory and immunization care. Moreover, abundance of overlapping buffers (2 km) representing areas of over service was found in two- third of villages for ambulatory and immunization services and in more than half of villages for delivery and inpatient care suggesting sub-optimal allocation of service delivery. However, such isodistance approach measuring ‘as the crow flies’ is predisposed to the overestimation of population served. Hence, estimating travel time adjusting for the type of environment being travelled like road network, physical barriers, landscape variation and elevation was undertaken subsequently.

**Table 2:** Results for proximity analysis: Percentage of villages within distance buffers.

	No Village within Buffer	One Village within Buffer	>1 Village within Buffer
**Ambulatory Care**			
**2 KM**	11.11	25.0	63.88
**5 KM**	0.5	4.44	95.55
**Delivery Points**			
**5 KM**	11.11	32.22	56.66
**10 KM**	Zero	3.88	96.11
**Inpatient Care**			
**5 KM**	12.22	34.44	53.33
**10 KM**	Zero	5.0	95.0

### 3.2 Accessibility analysis

**Fig 4:**
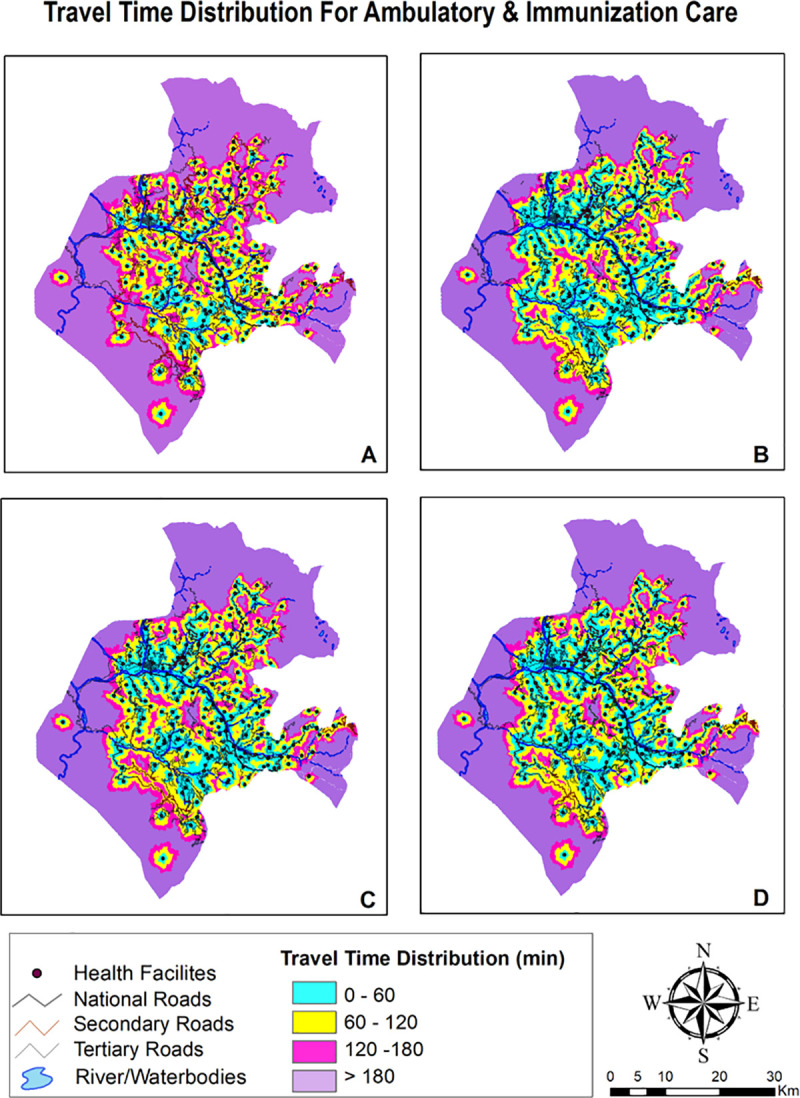
Mapping travel time grid for ambulatory & immunization care.

**Fig 5:**
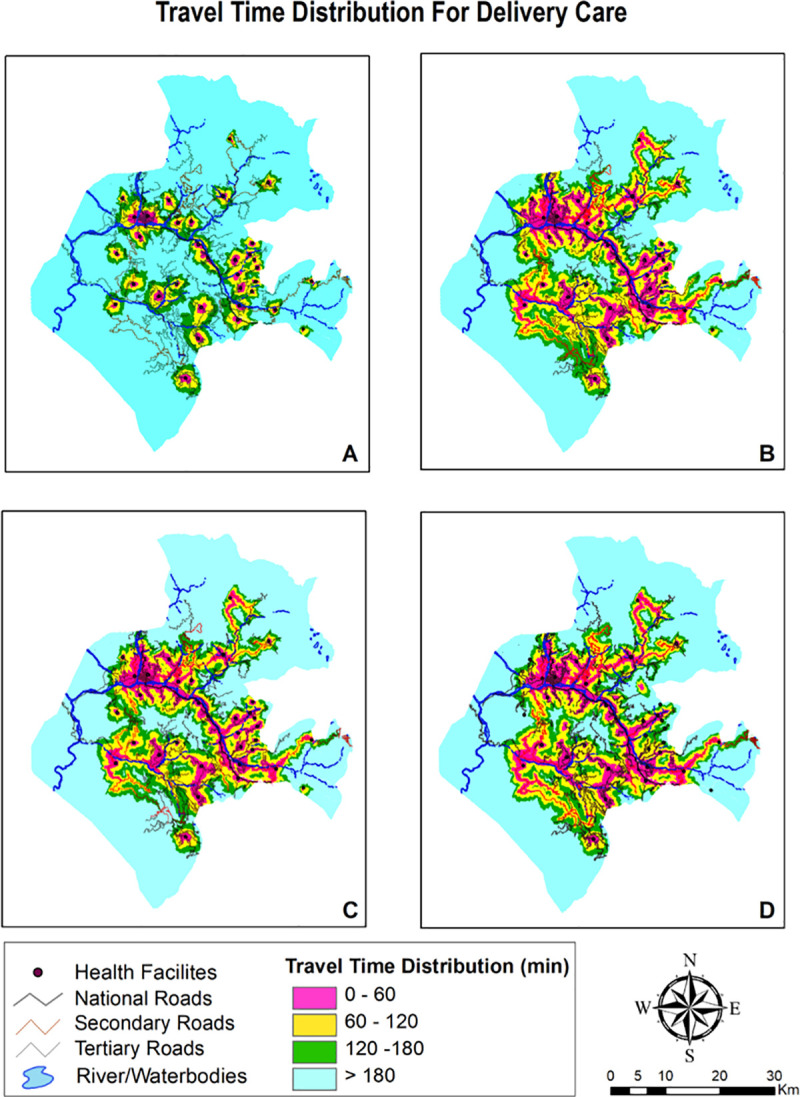
Mapping travel time grid for delivery care.

**Fig 6:**
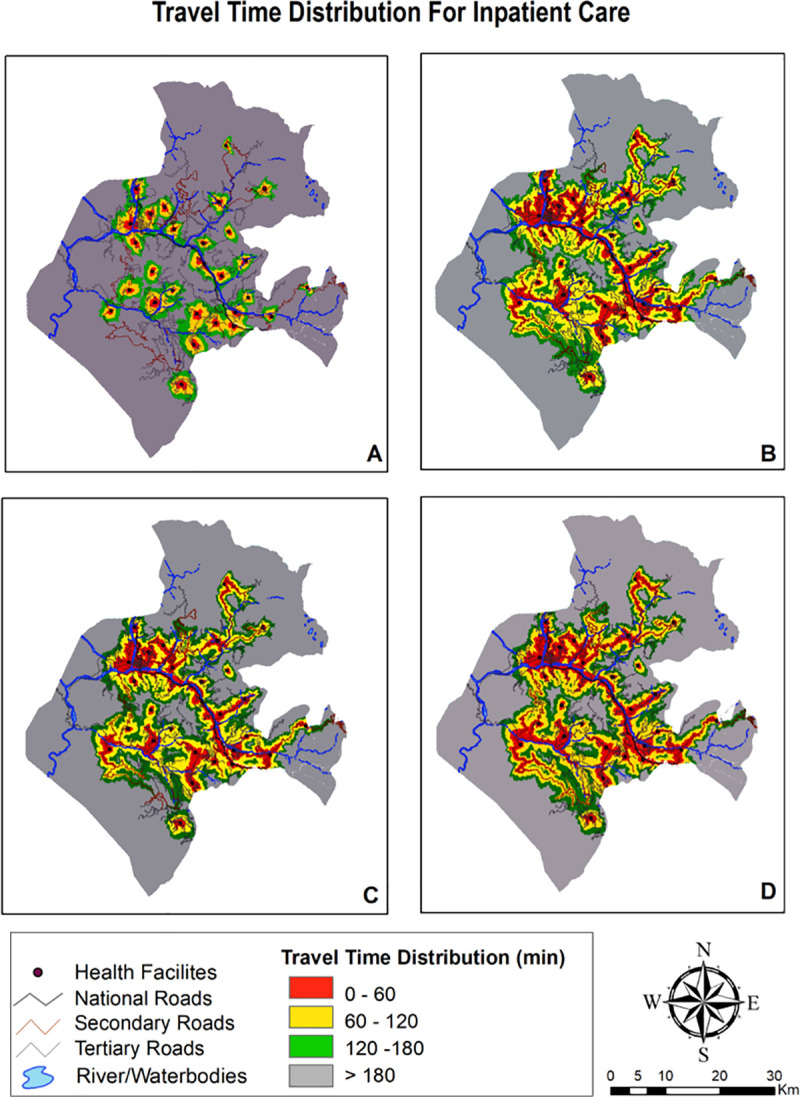
Mapping travel time grid for inpatient care.

The travel time distribution grid for four package of services and distinct travel time scenarios are summarized in Table 3 and visually depicted in Figs 4–6. The district wise percentage of unserved villages dwindled with faster means of transportation with the walking scenario having highest percentage of poor access followed by public transportation and lastly, privately owned motorized transportation. Maps providing gradient of travel time also explicates that spatial accessibility measures for different package of services is quite disparate. Time taken to commute to nearest facility for ambulatory and immunization was lower than inpatient and delivery care which is due to the fact that all facilities in the district are equipped for provisioning of ambulatory and immunization care, whereas only few facilities provide delivery and inpatient care.

**Table 3:** Percentage of villages in each travel time catchment by travel scenario.

	Walking	Walking and two wheeler	Walking & public transport	Walking & private vehicle
	**AMBULATORY AND IMMUNIZATION CARE**			
**Within an hour**	33.33	57.77	49.44	53.33
**Within 2 hours**	38.33	26.66	31.66	28.33
**Within 3 hours**	15.00	06.11	07.77	08.88
**More than 3 hours**	13.33	09.44	10.55	09.44
	**DELIVERY POINTS**			
**Within an hour**	13.33	30.00	30.62	32.77
**Within 2 hours**	17.22	33.33	25.00	25.00
**Within 3 hours**	23.88	15.00	18.33	20.00
**More than 3 hours**	45.00	21.66	29.44	22.22
	**INPATIENT CARE**			
**Within an hour**	07.77	27.22	23.88	27.22
**Within 2 hours**	11.11	32.77	25.55	25.55
**Within 3 hours**	17.22	17.77	20.00	23.33
**More than 3 hours**	63.33	22.22	30.55	23.88

#### Scenario 1

Walking scenario exhibited smallest accessible catchment area and travel distance covered within an hour was tethered to immediate vicinity. Even with adequate number of facilities providing care, median time taken to access ambulatory and immunization care as exhibited in Table 4 was found to be over 1 hour. Access to delivery and inpatient care points was found to be constrained with median time to reach these facilities as 2.2 hours and 2.7 hours respectively. Insurmountable barriers to access were evident as it takes more than 3 hours to reach delivery and inpatient care facilities for 45% and 63% villages.

**Table 4:** Median travel time to reach health facilities by travel scenario.

	Scenario 1	Scenario 2	Scenario 3	Scenario 4
**Ambulatory and Immunization Care**	1.4	0.8	1.0	0.9
**Delivery Care**	2.9	1.5	2.0	1.5
**Inpatient Care**	3.6	1.6	2.0	1.7

#### Scenario 2

Accessibility increased once travel by motorcycle was enabled. Median travel time vis a vis scenario 1 abated by one third for ambulatory and immunization care with 85% villages becoming accessible within 2 hour time. Time taken to travel for delivery and inpatient care also declined to more than half in this scenario. Travel by motorbike increases accessibility due to composite of a) Plausibility of motorbikes to run on certain land-cover surfaces and b) Existence of rudimentary road network compatible with motorcycle travel only.

#### Scenario 3

Public transport is slower mode vis. a vis. privately owned modes of travel as its less seamless and convenient. Interferences, fixed routes and time schedules, greater maneuvering issues and time lost at passenger stops renders this mode slower. Median time taken to reach ambulatory and immunization care was computed to be an hour, whereas, reaching delivery points and inpatient care takes an average of hour and half to two hours. In spite of enabling public transport in the modelling, around 50% villages remained outside the 2 hour realm.

#### Scenario 4

Owning a private four wheeler vehicle shrinks average travel time to less than an hour for ambulatory and immunization and one and half hour for delivery and inpatient facilities. Albeit, even the results for best scenario case (own private car) were dampened with half the area unable to access the ambulatory and immunization services within an hour of travel, whereas, it was much more faltered for delivery points and inpatient care with 42.2% and 47.2% area respectively being inaccessible within 2 hours of travel.

### 3.3 Geographic coverage analysis

**Fig 7:**
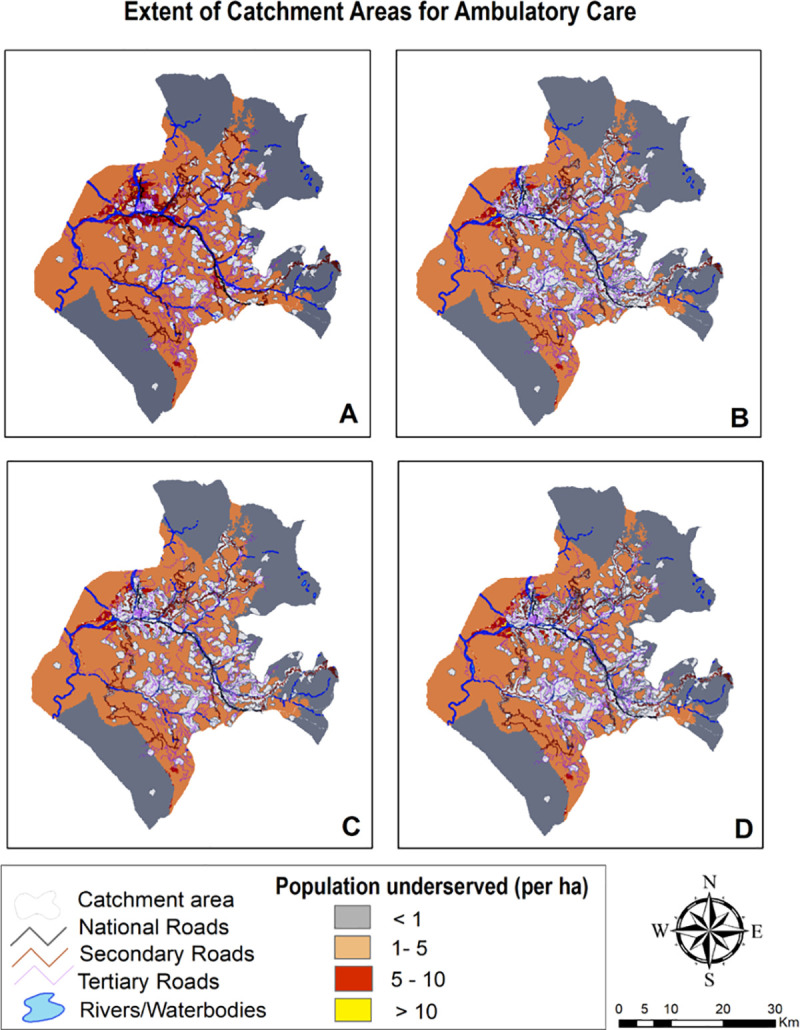
Mapping coverage for ambulatory & immunization care (1 hour time).

**Fig 8:**
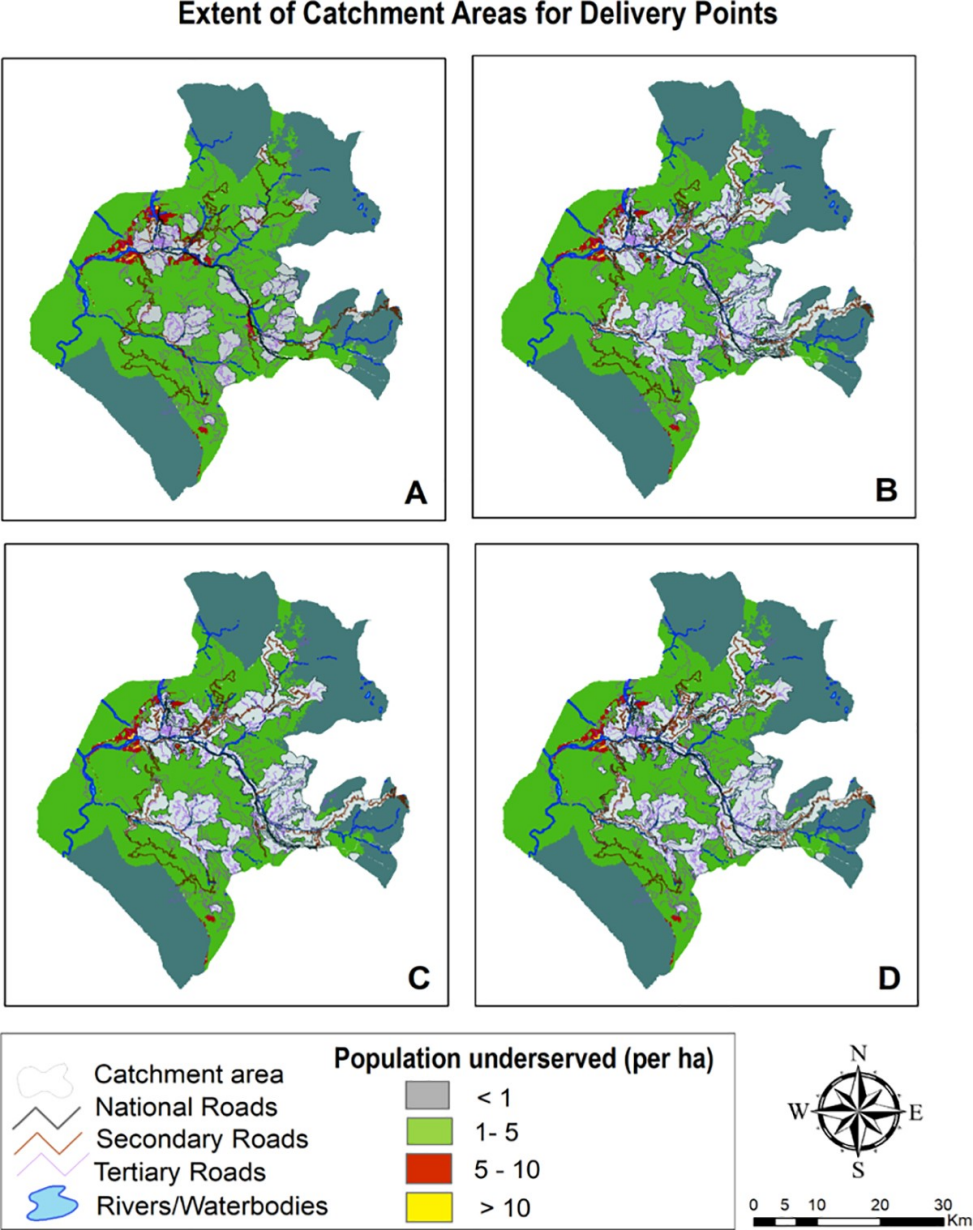
Mapping coverage for delivery care (2 hour travel time).

**Fig 9:**
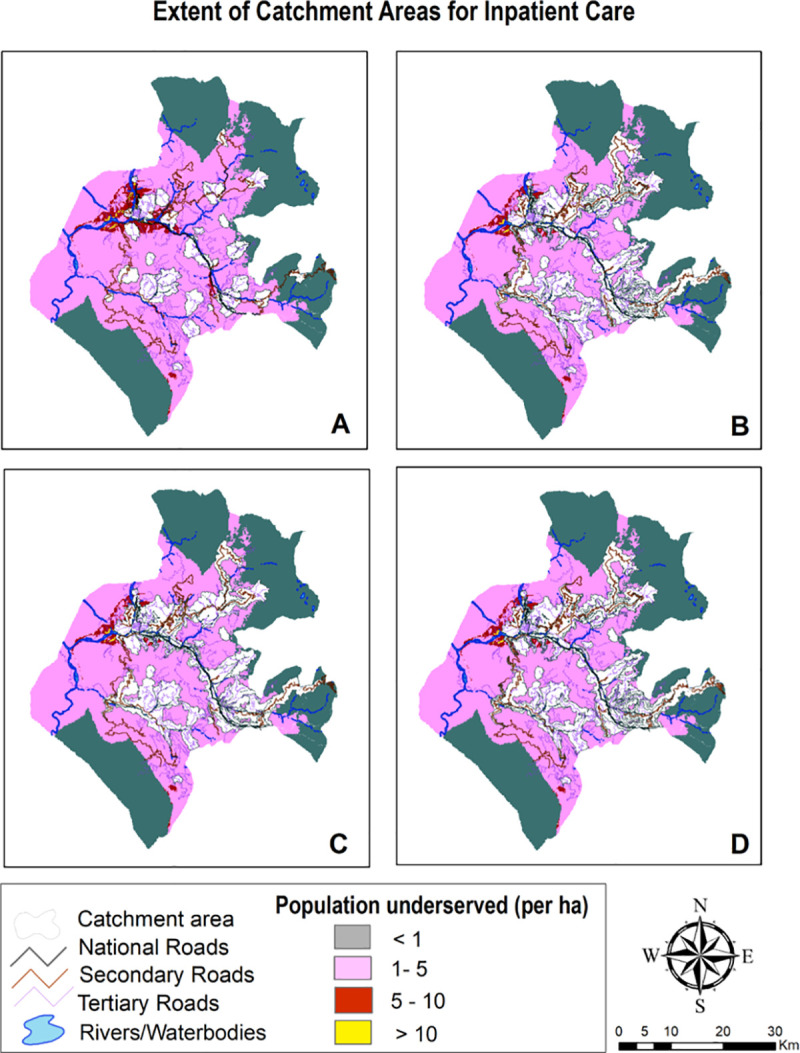
Mapping coverage for inpatient care (2 hour travel time).

Geographic coverage analysis conflating accessibility surfaces with settlements data extricated layer of catchment areas which is outlined in Figs 7–9 and surmised in Table 5. The pattern found in coverage analysis was in tandem with accessibility analysis with walking scenario enclosing miniscule proportion of population and proportion of population served increasing as motorized transportation was permitted in the model. Visualization of maps divulged that unserved population is more pronounced in peripheral areas with tenuous or rudimentary road network. For a given maximum travel time, proportion of served population was sensitive to mode of transportation.

**Table 5:** Distribution of population served in each travel time catchment by travel Scenario.

	Scenario 1		Scenario 2		Scenario 3		Scenario 4	
	1 hr	2 hrs	1 hr	2 hrs	1 hr	2 hrs	1 hr	2 hrs
***Ambulatory***								
**Percentage covered**	17.24	29.08	34.72	44.37	31.39	42.82	35.48	44.52
**Percentage uncovered**	82.76	70.92	65.28	55.63	68.61	57.18	64.52	55.48
**Facilities realizing maximum travel time**	31.81	13.43	30.43	13.43	25.40	16.13	32.35	12.89
**Facilities realizing maximum capacity**	67.57	86.48	70.27	86.48	74.51	83.78	67.57	87.02
***Delivery***								
**Percentage covered**	11.16	21.38	27.31	35.82	24.30	34.49	28.49	35.97
**Percentage uncovered**	88.84	78.62	72.69	64.18	75.7	65.51	71.51	64.03
**Facilities realizing maximum travel time**	67.64	41.17	52.94	14.70	50.0	26.47	50.0	17.64
**Facilities realizing maximum capacity**	32.35	58.82	47.06	85.29	50.0	73.52	50.0	82.35
***Inpatient***								
**Percentage covered**	6.03	16.91	23.56	34.91	18.02	33.64	27.88	35.06
**Percentage uncovered**	93.97	83.09	76.44	65.09	81.98	66.36	72.12	64.94
**Facilities realizing maximum travel time**	75.0	42.85	53.57	10.71	50.0	21.42	57.14	57.14
**Facilities realizing maximum capacity**	25.0	57.14	46.42	89.28	50.0	78.57	42.85	42.85

**Fig 10:**
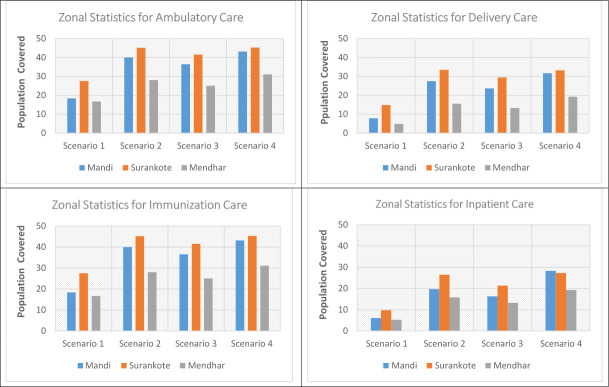
Zonal statistics for population coverage.

Also, vast swathes of unserved population area was congruous to less population density. When stratified at medical block level (Fig 10), it was revealed that block Surankote with secondary and relatively new road network and smaller population had modestly better accessibility, even though this block has higher elevation and has snow-fed fragment of land-cover. Conversely, block Mandi which exhibited least accessibility and greatest degree of remoteness is beset more with bridle tracks and larger population levels. Another feature underscoring block with least accessibility was low Pearson correlation coefficient between the set of travelling times and corresponding covered population within that time insinuating that population isn’t uniformly distributed while expanding outwardly from facility.

For walking scenario, only 17% population could be reached within an hour of ambulatory and immunization care and no significant improvement was found in coverage (29%) when travel time was extended to 2 hours. Moreover, due to paucity of facilities providing delivery and inpatient care, coverage was further contracted to 21% and 16% within 2 hour travel time. Facilities realizing maximum travel time was lower than facilitates realizing maximum capacity for ambulatory and immunization care; contrarily, greater number of facilities realized maximum travel time for delivery and inpatient care. Thus, facilities under capacity was highest for delivery and inpatient care with 67.64% and 75% facilities realizing their maximum travel time of an hour. Even with motorcycle scenario, more than half of population for ambulatory and immunization care and more than two-thirds for delivery and inpatient care remained underserved within 2 hour travel time. Facilities realizing maximum capacity exceeded facilities realizing maximum travel time of 2 hours that implied suboptimal capacities of facilities to provide care. Incongruously, if the maximum travelling time is reached before the population coverage capacity, the final extent of catchment area is only controlled by travelling parameters and health facilities can therefore be considered as underserved. Population density grid representing unserved population in public transport scenario was found to be contingent upon primary & secondary road network and built area. Again, the best case scenario with privately owned vehicle encompassed more than half of unserved population within 2 hour travel- time for all package of services.

### 3.4 Conflict & accessibility

For decades, the intricate linkage between external dimension (Indo-Pak conflict) and internal dimension (militancy in the area) leading to proxy wars and military skirmishes has perpetuated ordeal for border population having an abode in this area. The district has 60% of its population inhabiting border villages and has one fifth of the health facilities located in direct line of fire. Multitude of security disruptions through mortar shelling and firing and fear perpetuated by armed conflict is causing obstruction of access to health workers and patients, making the health system pregnable. Spatial accessibility gets even more constrained during the heightened border tensions, thereby, rendering the population to access facilities by walking only scenario. The movement of automated vehicle is not allowed during heavy exchange of fire; therefore, an alternative scenario of walking only was modelled for these border villages. Moreover, the speed of walking is slower during these circumstances as patients have to take an alternative route via ridges to dodge the bullets and splinters. After deliberating with locals and health workers, the walking speed for various classes of landscape and roads was reduced to half and scenario 1 was remodeled for border villages to capture travel time during conflict situation. In the alternate scenario of conflict, only 10% border villages were under 1 hour of travel time for ambulatory and immunization care; whereas just 7% and 5% border villages could access facilities for delivery and inpatient care respectively. The median travel time underscored unprecedented high range with 2 hours of travel time for ambulatory & immunization care and around 4 hours for delivery and inpatient care.

## 4. Discussion

In this analysis, investigation of geographic accessibility and spatial coverage of existing health facility network in district was conducted. The study demonstrated that integrating and analyzing spatial data by GIS technique can inform policy decisions for optimal resource allocation. Results of spatial modelling underscored the modal split leaning against walking as access was abysmal for all packages of care under this scenario. Pervasive gap between motorized and non-motorized mode and a modest gap between public and privately owned transport were accentuated in the study. These results are convergent with other studies conducted in similar rural and remote settings [17–20]. Coverage levels for delivery and inpatient care were inordinately low and were even lesser than African region. In a study conducted in sub Saharan Africa, it was conceded that only 7 out of 48 sub Saharan African countries had less than 50% population within 2-hr travel time of public emergency care hospital [21]. One of the reason of relatively poor inaccessibility is the fewer number of facilities providing delivery and inpatient care in our context. Adding a caveat; absenteeism, locked facilities, workforce shortage and poor quality perception leads to exacerbation of spatial inaccessibility. It is therefore, proposed to upgrade and reinvigorate already existing facilities especially primary health centers to become more responsive to delivery and inpatient care. Further, to attenuate the impediments arising from longer emergency medical services response times due to inaccessibility of hospitalization care, community workers could be trained as first responders to deliver basic emergency medical services.

Moreover, inaccessibility would be more pronounced if relative access to adequate transportation and affordability dimension in the local context is factored in. Our household survey (Additional dataset 2, Table 5) revealed that only 8% population had motorcycle ownership and merely 4.8% population possessed four wheeler (clustered around high density area), rendering majority of population vulnerable to walking only or public transportation scenario. Further, field observations suggested the frequency of public transport in the area to be exiguous. As a corollary, this translates to greater travel impedance for economically weaker sections and regions. Contextually, concerted efforts to bolster public transport especially for those with low income should be made to improve access. In the short run, geographic inaccessibility in ambulatory care can be circumvented by setting up telemedicine nodes in peripheral facilities as recommended in other studies as well [22–24] Hybrid model of telemedicine using both store and forward (asynchronous) as well as real time (synchronous) connectivity within Hub and Spoke framework can be introduced to provide accessible, affordable and quality healthcare. Subcenters in remote catchment areas acting as spokes can be transformed to e-subcenter where telemedicine nodes can be established connecting them via satellite link with hub acting as specialty nodes. Moreover, targeting delivery care, programmatic intervention establishing affordable maternity waiting homes during last weeks of pregnancy in remote catchment areas which are more than two hours away from delivery points should be considered. However, healthcare accessibility can be augmented by systems approach embodying synergistic interventions at the community, facility and health systems level designed to decrease travel time to care and increase access to motorized transportation. [25].

Comparison of theoretical estimates predicted by the model for median travel time and empirically reported median travel time (Additional dataset 2, Table 4) revealed slight overestimation by theoretical estimates for walking only scenario to access ambulatory care. Conversely, for travel scenario with public transportation, theoretical estimates underestimated the reported statistic by 9.5% for ambulatory care and 22% for inpatient care. This finding converges with another study conducted in rural South Africa which revealed theoretical model overestimating the walking time by 6% vis. a vis. reported time and larger variability in public transport model [26].This can be attributed to the fact that our modelling predictions does not factor for longer waiting time and exiguous frequency of public transportation in remote areas. Also, care seeking for inpatient care is disproportionately higher in higher order and inter-district hospitals, thereby, amplifying the median reported time for inpatient care. Furthermore, using village centroids rather than exact location of the households reduced the precision of the estimates as in setting such as ours households are dispersed over the village.

Another dimension to be highlighted is that inaccessibility gets festered during security disruptions with cross border firing and shelling episodes owing to disturbed border situation. Multitude of security disruptions through shelling, firing and fear perpetuated by armed conflict causes obstruction of access rendering health system pregnable and difficult to access. In a conflict ridden district of Kashmir, a study revealed that unserved population was twice as large during scenario of political disruption, curfews, strikes and communication blockades etc. vis a vis normal times [27]. Similar results were disseminated in yet another study in South Sudan where 22% population had walking time to nearest clinical service of more than 5 hrs and the scenario was much worse in specific counties where current civil war started affecting already fragile health system contributing to closure of many facilities [28].

Although, study undertook a quiet nuanced exercise of modelling average travel times, such average measures of travel precludes complex and interacting variables involved in accessing care. The model is invariant to temporal variability in transport and doesn’t include impact of seasonal and weather conditions cogently. Study can be further augmented by combining temporal variation with multiple modes of travel producing complex multidimensional array of access scenarios, thereby, supporting dynamic interactivity. Further, time taken to reach facilities is influenced by sequence of decisions and actions. Decision to seek care upon onset of illness is idiosyncratic to time preferences with ideal scenario being decision taken promptly. However, when care is sought, actual time to access facility is likely to be greater than modelled travel time. Hence, agent based modelling application exploring complexity in health behavior could be synthesized with the modelling approach in this study.

Another argument in the study stems from parsimonious assumption that patients travel to nearest health facilities and travel invariably along optimum paths in terms of total travel time. The estimated travel time is therefore assumed to be illustrative of real travel times. While some members of population may use alternative paths due to habits, social factors, environmental or surface conditions, severity of illness or other factors; the least cost approach still reflects the overall mode in which people tend to travel [18]. Besides, travel impedance to nearest provider has been assumed to be a good measure of spatial accessibility for rural areas, where provider choices are very limited and nearest provider is also the most likely to be used. This resonates with our context as in our region, 92% population resides in rural areas having presence of only public providers with only handful of private providers in the district predominantly concentrated in the town area. Our household survey further divulged that the decision to consult private provider was guided by quality considerations rather than geographical accessibility (Additional dataset 2, Figs 5–8). However, for ambulatory care, 65% people who circumvented public facilities cited geographical inaccessibility as the major reason for seeking care from informal providers like pharmacies and traditional healers.

Coverage or service areas in this study were delineated through the creation of buffer zones at various impedance levels through Euclidean (straight-line) distance and travel time analysis along the raster. The results were very sensitive to choice of method, since the distance method over-estimated the population covered. Overestimation by the Cartesian distances specifically Euclidean distance method, which is most widely used in the literature is being underscored by many studies [29,30]. In a similar setting, modelling distances travelled to government health facilities revealed that Euclidean distance model overestimates population within 1 hour of health facility by 19% in Kenya [8]. Another study conducted in Guatemala, divulged that coverage estimates based on road network analysis were 18% lower than the crow flies estimates [31]. Such divergence could be explained by the usage of isochrone maps in Euclidean measures which are based on the premise that everyone within threshold area have same probability of access. This assumption is circumnavigated in literature by using distance decay function with more realistic assumption that facility serves specific catchment area and the draw on the population to those services decreases with increasing distance from the facility. However, this method isn’t bereft of limitations as there’s an ambiguity in choosing appropriate distance decay function with appropriate impedance coefficient. Also, planimetric mapping is not suitable for rough and mountainous terrains whereas, raster-based travel time surfaces allows for landscape constraints and serve as a faster, replicable and scalable alternative. Hence, we estimated the travel time in conjunction with travel distance as it is a better indicator of travel impedance in our area where inaccessible terrain and multiple transportation modes to reach care translates to longer travel times for relatively short distances.

The scope of study can be extended to analyze how spatial accessibility varies across the spectrum of disease. Also, current modelling was based on the residence of patients and doesn’t take into account the location of their work. There is further scope of refining the spatial accessibility estimates for relative high preference for higher order facilities by patients when pairs of government health facilities are located adjacently. Such adjustments are made in some studies by developing *transect* algorithm to quantify how patients respond to competing array of health service providers [8,32]. Additionally, more complex readjustments for socio-economic determinants of spatial access to health services can be propounded in the extension of this work.

## 5. Conclusions

Our study is conducted in one of the geographically isolated and conflict prone region in India where topographical adversities and institutional factors impede access to healthcare services. The study is a comprehensive application of AccessMod 5.0 to assess physical accessibility of current health system enabling identification of gaps and weaknesses w.r.t to reaching target populations. The modelling exercise highlighted the need for an evidence based spatio-temporal approach to public health for increased transparency in decision making and better understanding of geographical impediments in achieving the goal of Universal Health Coverage. The methodological framework adapted in this study can be generalized to both difficult and normal settings as well. Results indicated relatively adequate access by distance measure, however, travel time measures unraveled inaccessibility when modelled via more nuanced raster approach. Motorized transportation improved the geographic accessibility to some extent, with private ownership of automated vehicle augmenting the coverage the most. However, accessibility and coverage remained exiguous even in the best scenario. Low coverage was attributed to the unprecedented physical barriers and topography constraints and also, less/suboptimal capacity of facilities which is incommensurate with demands of population.

Based on the results, these suggestions are needed to be implored- a) Bolstering the existing health care network especially for the provision of delivery and inpatient care as facilities are functioning under-capacity and warrants focus on augmenting capacity of existing facilities so that larger population could be covered within set travel time if their coverage capacity is extended and readiness is improved. In our resource constraint setting, a better spatial distribution of service availability in currently placed facilities is suggested to improve access in terms of increased availability, accessibility, affordability, acceptability and accommodation b) Training community workers as first responders in short run in the border villages experiencing inordinately high travel time due to military disruptions and propelling inter-departmental cooperation and interventions in the medium to long run is recommended and c) Setting up of telemedicine and mobile health units in hard to reach pockets should be strategized to overcome the greater travel impedance. Scope of this study can be extended to analyze how spatial accessibility varies temporally for various seasons and across the spectrum of disease. Further, modelling exercise can be extended to map spatial accessibility of patients based on their location of work and integrating the data on poverty and population structure to create more robust indicator supporting planning for delivery of services.

## Supporting information

S1 File Additional dataset 1. (XLSX)

S2 File Additional dataset 2 findings from household survey. (DOCX)
