# Supplementary material for: Geographical accessibility and spatial coverage modelling of public health care network in rural and remote India
Source: PLoS One. 2020 Oct 21;15(10):e0239326. doi: 10.1371/journal.pone.0239326 (PMC7577445; doi:10.1371/journal.pone.0239326)
Supplement: S2 File — (DOCX) [file pone.0239326.s002.docx]

**Additional Dataset 2 FINDINGS FROM HOUSEHOLD SURVEY**

| ***TABLE 1: DESCRIPTIVES OF THE SAMPLED POPULATION*** | | | |
| --- | --- | --- | --- |
|  | ***Mandi*** | ***Surankote*** | ***Mendhar*** |
| *Household screened* | *3000* | *2200* | *2800* |
| *Household selected* | *600* | *440* | *560* |
| ***HH selection by service package*** | | | |
| *Ambulatory care* | *120* | *88* | *112* |
| *Inpatient care* | *240* | *176* | *224* |
| *Delivery care* | *120* | *88* | *112* |
| *Others* | *120* | *88* | *112* |
| ***Individuals selected by service package*** | | | |
| *Ambulatory care* | *216* | *177* | *196* |
| *Inpatient care* | *289* | *199* | *262* |
| *Delivery care* | *120* | *88* | *112* |
| *Immunization care* | *293* | *228* | *259* |
| *Others* | *2200* | *1445* | *1954* |
| **Total** | *3418* | *2437* | *3109* |
| ***Sector*** |  |  |  |
| *Rural* | *100* | *100* | *100* |
| *Urban* | *0* | *0* | *0* |
| ***Gender*** |  |  |  |
| *Male* | *51.8* | *49.4* | *52.3* |
| *Female* | *48.2* | *50.6* | *47.7* |
| ***Education Level*** |  |  |  |
| *No education* | *30.1* | *29.7* | *29.9* |
| *Primary education* | *34.6* | *34.8* | *33.4* |
| *Secondary education* | *18.3* | *19.5* | *19.2* |
| *Tertiary education* | *17.3* | *16.0* | *16.5* |
| ***Employment Status*** |  |  |  |
| *Regular wage/salary* | *8.9* | *10.8* | *9.3* |
| *Self-employed/Agriculture* | *40.4* | *39.2* | *37.1* |
| *Casual Labour* | *38.3* | *36.8* | *38.9* |
| *Unemployed* | *12.4* | *13.2* | *14.7* |
| ***Household size*** |  |  |  |
| *<5 members* | *21.4* | *24.5* | *19.7* |
| *>5 members* | *78.6* | *75.5* | *80.3* |
| ***Median Age*** | *23.4* | *24.5* | *23.8* |
| ***Religion*** |  |  |  |
| *Muslim* | *91.5* | *97.5* | *94.6* |
| *Hindu* | *7.0* | *2.5* | *3.6* |
| *Others* | *1.5* | *0* | *1.8* |
| ***Monthly Per Capita Income(INR)*** | *1420* | *1395* | *1440* |
| ***Household Asset Index*** |  |  |  |
| *Poorest* | *20.2* | *20.1* | *19.7* |
| *Second* | *20.4* | *19.9* | *20.4* |
| *Middle* | *19.7* | *20.0* | *20.2* |
| *Fourth* | *19.8* | *20.1* | *19.9* |
| *Richest* | *19.9* | *19.9* | *19.8* |
| ***Major source of drinking water*** |  |  |  |
| *Tap* | *32.9* | *31.2* | *42.3* |
| *Tube-well/Hand pump* | *7.8* | *5.9* | *15.2* |
| *Pond/spring reserved for drinking* | *40.6* | *44.3* | *28.6* |
| *River/canal* | *19.7* | *18.6* | *13.9* |
| ***Household structure*** |  |  |  |
| *Kutcha* | *33.7* | *39.5* | *38.3* |
| *Pucca* | *19.8* | *17.1* | *18.4* |
| *Semi-pucca* | *46.5* | *43.4* | *45.3* |
| ***Marital Status*** |  |  |  |
| *Never Married* | *54.3* | *54.5* | *56.3* |
| *Currently Married* | *36.4* | *35.6* | *34.5* |
| *Widowed* | *8.2* | *8.3* | *7.9* |
| *Divorced/Separated* | *1.1* | *1.6* | *1.3* |
| ***Type of Latrine*** |  |  |  |
| *No latrine* | *35.7* | *32.0* | *31.6* |
| *Pit* | *19.2* | *18.6* | *17.0* |
| *Septic tank/Flush system* | *45.1* | *49.4* | *51.4* |
| ***Type of drainage*** |  |  |  |
| *No drainage* | *81.7* | *79.5* | *78.6* |
| *Open kutcha* | *13.0* | *14.5* | *13.9* |
| *Open pucca* | *5.3* | *6.0* | *7.5* |
| ***Primary source of energy for cooking*** |  |  |  |
| *Firewood and chips* | *45.1* | *47.7* | *43.7* |
| *Gobar gas/Dung cake/Coal* | *15.8* | *12.3* | *10.2* |
| *Kerosene* | *12.3* | *13.4* | *15.3* |
| *LPG* | *26.8* | *26.6* | *30.8* |

| ***Table 2: Different Travel Scenarios/Mode of transportation used by Ailing to reach facility (in %)*** | | | | |
| --- | --- | --- | --- | --- |
| **Travel Scenario** | **Ambulatory Care** | **Inpatient Care** | **Delivery Care** | **Immunization Care** |
| Walking | 28 | 5 | 4 | 40 |
| Two- wheeler | 8 | 3 | 0 | 5 |
| Public Transport | 4 | 10 | 6 | 11 |
| Private vehicle | 7 | 9 | 8 | 3 |
| Walking +Two-wheeler | 6 | 14 | 0 | 9 |
| Walking + Public transport | 35 | 26 | 17 | 27 |
| Walking+ Private vehicle/rental | 12 | 29 | 57 | 5 |
| Ambulance | 0 | 4 | 8 | 0 |
| Total | N=590 | N= 750 | N= 320 | N= 780 |

| ***Table 3: Average distance travelled by Ailing to reach point of care in different scenarios (in kms)*** | | | | |
| --- | --- | --- | --- | --- |
| **Travel Scenario** | **Ambulatory Care** | **Inpatient Care** | **Delivery Care** | **Immunization Care** |
| Walking | 3.5 | 7 | 3 | 2.5 |
| Two- wheeler | 10 | 15 | NA | 3 |
| Public Transport | 13.5 | 25 | 15 | 4 |
| Private vehicle/rental | 16 | 45 | 29 | 3 |
| Walking +Two-wheeler | 11 | 16 | NA | 3 |
| Walking + Public transport | 9.5 | 26 | 14 | 4.5 |
| Walking+ Private vehicle/rental | 18 | 55 | 28 | 4 |

| ***Table 4 : Median time taken by Ailing to reach point of care (in hours)*** | | | | |
| --- | --- | --- | --- | --- |
| **Travel Scenario** | **Ambulatory Care** | **Inpatient Care** | **Delivery Care** | **Immunization Care** |
| Walking | 1.2 | 0.6 | 1.0 | 0.4 |
| Two- wheeler | 0.5 | 0.5 | NA | 0.3 |
| Public Transport | 0.9 | 2.5 | 1.5 | 0.7 |
| Private vehicle/rental | 0.8 | 2.7 | 1.2 | 0.9 |
| Walking +Two-wheeler | 0.8 | 1.4 | NA | 0.7 |
| Walking + Public transport | 1.1 | 2.5 | 1.8 | 1.0 |
| Walking+ Private vehicle/rental | 0.9 | 2.0 | 1.6 | 0.6 |

| ***Table 5: Ownership of vehicles in the survey households disaggregated by Medical blocks in district*** | | | |
| --- | --- | --- | --- |
|  | **Mandi** | **Surankote** | **Mendhar** |
| **No vehicles** | 89.3 | 90.0 | 88.2 |
| **Two wheeler only** | 5.8 | 5.2 | 7.0 |
| **Four - wheeler only** | 3.0 | 2.7 | 3.0 |
| **Two wheeler and Four wheeler** | 1.8 | 2.0 | 1.8 |
| *Total number of households* | 600 | 440 | 560 |
